# Supplementary material for: Situation awareness of emergency response centre personnel during chemical incidents: an interview study in a Swedish context
Source: BMJ Open. 2023 Jun 14;13(6):e071347. doi: 10.1136/bmjopen-2022-071347 (PMC10277125; doi:10.1136/bmjopen-2022-071347)
Supplement: Supplementary data [file bmjopen-2022-071347supp001.pdf]

Interview guide: Situation awareness, Emergency Response Centre personnel

General information about the participant:

- How old are you?
- How many years have you worked at the Emergency Response Centre?
- Are you an operator/rescue service dispatcher/emergency medical service dispatcher?
- Have you previously worked with something that can be relevant considering that the interview is about chemical incidents? If yes – for how many years?
- What kind of education do you have?

Interview questions:

1. Can you describe a situation where you received an emergency call regarding an incident, immediately or later classified as a chemical incident, where there was a risk of people being injured or dying?
    a. What made you understand that it was more than an ordinary incident – an incident with chemicals involved when there was a risk of casualties?
        i. Was it something the caller said?
        ii. How did you find out the extent of the incident, for example if there were several people injured?
        iii. Was it evident from the start that it was a chemical incident with a risk for casualties?
    b. When you understood that it was a chemical incident with a risk for casualties, did you have access to an interview guide or other guide?
        i. What made you choose a specific interview guide?
            1. Do you ever need ask other questions than those in the interview guide? If yes – why?
            2. If you needed to ask other questions than those in the interview guide, whom did you ask and what did you ask?
        ii. Did you receive information about which chemical substance was involved?
            1. How do you generally receive information about which chemical substance is involved? / How do you usually do to receive information about which chemical substance is involved?
            2. Depending on the chemical substance, how did you have to adjust your questions in order to know how you should act?
    c. Did you consider that the incident could become dangerous for more people? /escalate?
        i. Why? / why not?
        ii. How did you think the situation could develop?
            1. Before the emergency organizations arrived?
                a. Do you manage the emergency calls about chemical incidents differently depending on the lead time, e.g., if

the incident is in a sparsely populated area or quite close by?

2. When the emergency organizations were at the incident site?
3. When the emergency organizations had left the incident site? (Is there a continued risk?)
    a. During major chemical incidents, the incident site can be polluted/unsafe for a long time - do you have continual preparedness for several people being injured at the same incident site?

d. What decisions did you make?
    i. What information did you give to the caller, what should he/she do?
        1. What do you think of the dilemma of safety versus saving lives?
    ii. How did you know which resources needed to be dispatched?
        1. Are there dispatch plans?
    iii. Is there a particular order in which you contact the emergency organizations? If yes – does this differ depending on if it is a chemical incident with the risk for casualties?
    iv. How do you inform the emergency organizations that it is a chemical incident with the risk for casualties?
    v. Which information do you expect the emergency organizations at the incident site to send to you?
    vi. How does the information you receive from the emergency organizations at the incident site influence your actions?
    vii. Do the decisions you take differ depending on the chemical substance involved?
    viii. Do your decisions differ depending on if you suspect an antagonistic incident and not a regular incident?

General questions to ask after the participants have described their experiences

1. Is there anything regarding emergency calls about chemical incidents with the risk for casualties that you consider challenging? - What? Why? / Why not?
    a. Do you need the support of a back office/ other support during chemical incidents? Do you sit together?
    b. Are there occasions when you feel that the incident is worse than what the caller thinks? – In that case, what do you do?
2. Are there situations when you more often consider that it can be a chemical incident with the risk for casualties?
    a. If an alarm comes from a Seveso facility, for example, does this influence how you think about the incident?
        i. Is there any extra information that you would ask for?
    b. Depending on if it is a citizen that makes the emergency call, a professional working at a Seveso facility, or a heavy goods vehicle driver, do you think differently?
    c. If an electric bus start burning, how do you proceed?
        i. Are there special tasks you need to perform?
        ii. How do you think the situation could develop if this happened in a tunnel/residential area?

3. If you receive an emergency call that suggests that the person's symptoms could be a consequence of a chemical incident, which information would you need to be certain?
    a. If several emergency response centre operators receive calls about persons with the same symptoms, how would you understand if it was the same chemical incident?
    b. During chemical incidents the symptoms does not always present at once. Do you usually take this into consideration when you make decisions?
4. How do you work with the operator/dispatchers during joint emergency calls during chemical incidents with the risk for casualties? Is there any difference compared to other major incidents?
    a. How do you ensure that all persons in the joint emergency call have the same information about the chemical incident?
5. What responsibility do you have when you have dispatched the emergency organization units and they have arrived at the incident site?
    a. Do you need to be prepared to adjust the emergency organization units depending on how serious the incident is and how many injured there are?
    b. If, during a chemical incident, people in the emergency organization units would become injured due to lacking information, what would you think of such an incident?
6. Is there anything which we have not talked about that you want to bring up? Do you have any questions?
