# Supplementary material for: Situation awareness of emergency response centre personnel during chemical incidents: an interview study in a Swedish context
Source: BMJ Open. 2023 Jun 14;13(6):e071347. doi: 10.1136/bmjopen-2022-071347 (PMC10277125; doi:10.1136/bmjopen-2022-071347)
Supplement: Author's manuscript [file bmjopen-2022-071347.draft_revisions.pdf]

BMJ Open

BMJ Open is committed to open peer review. As part of this commitment we make the peer review history of every article we publish publicly available.

When an article is published we post the peer reviewers' comments and the authors' responses online. We also post the versions of the paper that were used during peer review. These are the versions that the peer review comments apply to.

The versions of the paper that follow are the versions that were submitted during the peer review process. They are not the versions of record or the final published versions. They should not be cited or distributed as the published version of this manuscript.

BMJ Open is an open access journal and the full, final, typeset and author-corrected version of record of the manuscript is available on our site with no access controls, subscription charges or pay-per-view fees (http://bmjopen.bmj.com).

If you have any questions on BMJ Open's open peer review process please email info.bmjopen@bmj.com

BMJ Open

Situation awareness of Emergency Response Centre personnel during chemical incidents – an interview study

Journal:	*BMJ Open*
Manuscript ID	bmjopen-2022-071347
Article Type:	Original research
Date Submitted by the Author:	23-Dec-2022
Complete List of Authors:	Karlsson, Sofia; Umeå Universitet Medicinska fakulteten, Department of Surgical and Perioperative Sciences, Surgery<br>Gyllencreutz, Lina; Umea University Department of Surgical and Perioperative Sciences, Surgery
Keywords:	ACCIDENT & EMERGENCY MEDICINE, Telemedicine < BIOTECHNOLOGY & BIOINFORMATICS, QUALITATIVE RESEARCH

SCHOLARONE™
Manuscripts

I, the Submitting Author has the right to grant and does grant on behalf of all authors of the Work (as defined in the below author licence), an exclusive licence and/or a non-exclusive licence for contributions from authors who are: i) UK Crown employees; ii) where BMJ has agreed a CC-BY licence shall apply, and/or iii) in accordance with the terms applicable for US Federal Government officers or employees acting as part of their official duties; on a worldwide, perpetual, irrevocable, royalty-free basis to BMJ Publishing Group Ltd ("BMJ") its licensees and where the relevant Journal is co-owned by BMJ to the co-owners of the Journal, to publish the Work in this journal and any other BMJ products and to exploit all rights, as set out in our licence.

The Submitting Author accepts and understands that any supply made under these terms is made by BMJ to the Submitting Author unless you are acting as an employee on behalf of your employer or a postgraduate student of an affiliated institution which is paying any applicable article publishing charge ("APC") for Open Access articles. Where the Submitting Author wishes to make the Work available on an Open Access basis (and intends to pay the relevant APC), the terms of reuse of such Open Access shall be governed by a Creative Commons licence – details of these licences and which Creative Commons licence will apply to this Work are set out in our licence referred to above.

Other than as permitted in any relevant BMJ Author's Self Archiving Policies, I confirm this Work has not been accepted for publication elsewhere, is not being considered for publication elsewhere and does not duplicate material already published. I confirm all authors consent to publication of this Work and authorise the granting of this licence.

For peer review only

Situation awareness of Emergency Response Centre personnel during chemical incidents – an interview study

Karlsson, Sofia[a]; Gyllencreutz, Lina*[a]

a. Department of Surgical and Perioperative Sciences, Surgery, Umeå University, 901 87 Umeå, Sweden

*Corresponding author, lina.gyllencreutz@umu.se

Word count: 5340

Tables: 1

Figures: 0

ABSTRACT

Objectives If a chemical incident occurs, the Emergency Response Centre personnel are the first that are notified. They need to quickly attain situation awareness, based on the information from the caller, in order to dispatch the correct emergency units. The aim of this study is to examine the situation awareness of the personnel working at Emergency Response Centres - how they perceive, comprehend, project and act during chemical incidents.

Methods Semi-structured individual interviews with 12 participants from the Swedish Emergency Response Centres were performed. The interviews were analysed with qualitative content analysis.

Results Three categories of responses were identified. Responses focused on the complexity of identifying chemical incidents, the importance of ensuring the safety of citizens and personnel of emergency organizations, and the situation-based dispatch of organizations.

For peer review only - http://bmjopen.bmj.com/site/about/guidelines.xhtml

For peer review only

Conclusions The correct identification of the chemical incident and the involved chemical by the Emergency Response Centre personnel is necessary in order to notify, inform and dispatch the correct units, as well as to ensure the safety of citizens and emergency personnel. In order to potentially increase the situation awareness of the Emergency Response Centre personnel, a number of measures are suggested: increasing public knowledge, creating a joint chemical incident database and holding joint reflection seminars for the personnel of the Emergency Response Centres and emergency organizations.

STRENGTHS AND LIMITATIONS OF THIS STUDY

- This study contributes to the research area of situation awareness, combining theory with the management of chemical incidents by the personnel of emergency response centres. To the authors' knowledge, few researchers have studied this nexus before.
- A strength of the study is the geographical diversity of the participants, as well as their differing professional roles. This makes the study more comprehensive.
- A limitation of the study is that, due to a diverse system, the Swedish Emergency Response Centre personnel have different roles, responsibilities and education, depending on where in the country they operate.
- The interviews were performed via Zoom or Teams, which could have influenced the quality of the interviews. However, the interviews were rich in content and about one hour in length, which gave plenty of material to analyse.

INTRODUCTION

Worldwide, large quantities of chemical substances are produced, transported and used, making incidents probable (1, 2). Incidents can result from e.g. human error or poor maintenance of equipment used for manufacturing and storage (3). Incidents can include fires, explosions, leaks and spills, and structural collapse (3). Chemical incidents involving the

For peer review only - http://bmjopen.bmj.com/site/about/guidelines.xhtml

uncontrolled release of toxic substances can potentially lead to injury or death of exposed persons, as well as harm the environment (1). A previous chemical incident that killed and injured many people was the Beirut port explosion in Lebanon on 4 August 2020 (4). However, most chemical incidents are small or medium-sized, and generally involve few killed or injured persons (1, 3), here exemplified by a couple of Swedish chemical incidents that injured about 10 persons respectively (5, 6).

Internationally as well as in Sweden, when an incident occurs, the first point of contact with the emergency organizations is often the Emergency Response Centres (ERC). The ERC operator is tasked with gaining as much relevant information from a caller as possible, and with determining which response is required (7). Dispatchers are connected to the call to inform and dispatch the correct emergency organization units (7-9). The ERC personnel swiftly manage a large quantity of information of varying quality from different sources, such as the general public or on-site emergency personnel, in order to make decisions in a dynamically changing situation (10). Thus, in order to do this, ERC personnel utilise their situation awareness, which means they perceive elements in the environment, comprehend the current situation and project its future status (11). The ERC personnel form a mental picture of the situation, where they use their experience and intuition in order to decide how to properly deal with a call (12). Because every call is unique, ERC personnel must be sensitive listeners, and should be able to reformulate their questions depending on the situation, balancing their use of the interview guide and their common sense and intuition (13, 14). However, an initial lack of information about the incident can result in injured first responders, as in the case of the release of anhydrous ammonia from a farm tractor towing a 2-ton ammonia tank experiencing mechanical failure in Illinois, USA (15). The ERC personnel initially reported the incident as a car fire, which led to several first responders being unaware of the danger. A number of responders were thus exposed to the gas, and some

For peer review only

For peer review only - http://bmjopen.bmj.com/site/about/guidelines.xhtml

later experienced symptoms of illness (15). Thus, the ERC personnel's situation awareness, related to their ability to identify the incident and predict medical outcomes for those affected, is highly important in chemical incidents.

The literature states that further study is needed on the challenges of situation awareness and behaviours used by the ERC personnel to overcome those challenges (16). Further, few studies focus solely on the ERC personnel's experiences of chemical incidents. Because of the prevailing danger during chemical incidents for the affected persons and responders alike, ERC personnel need to be able to manage the incoming information. This is especially true during major chemical incidents, when many persons may potentially be injured. Thus, the aim of this study is to examine the situation awareness of the personnel working at Emergency Response Centres - how they perceive, comprehend, project and act during chemical incidents.

METHOD

Setting

In Sweden, the personnel at the Emergency Response Centre are responsible to answer the national emergency number 112 and interview the callers. Based on the information they receive, personnel should then dispatch and coordinate the emergency medical service and rescue service units. The ERC are also collaborating with other organizations, such as the police (8, 17). In some Swedish regions, registered nurses perform the medical interview instead of an ERC operator (8, 18).

Participants

Information about the study was provided to five managers of the Emergency Response Centres located in various parts of Sweden. The managers notified their personnel of the

For peer review only - http://bmjopen.bmj.com/site/about/guidelines.xhtml

study, and interested personnel with experience of chemical incidents were invited to participate. In total, 12 respondents participated. Nine of the participants were women and three were men. Their ages ranged from 32 to 63 years (with a mean age of 43), and their work experience at the emergency response centres varied from 2 to 32 years (with a mean length of eight years). The participants worked as ERC operators (whereof some were registered nurses), rescue service dispatchers, or emergency medical service dispatchers. Some of the participants had just one of these roles, while several of them rotate between roles - for example, variously working both as an ERC operator and a rescue service dispatcher.

Patient and public involvement

Patients or the public were not involved in the design, conduct, reporting or dissemination of this research.

Data collection

The semi-structured interview guide (see online supplemental file 1) was inspired by the theory of situation awareness, applying the concepts of perception of elements in the current situation, comprehension of the current situation, projection of future status and decision-making (11) to the ERC personnel's work during chemical incidents. The opening question was "Can you describe a situation where you received an emergency call regarding an incident, immediately or later classified as a chemical incident, where there was a risk of people being injured or dying". Many of the interview questions referred back to the described incident and situation awareness regarding that incident. For example, one question dealt with what it was during the emergency call that made personnel understand that a chemical incident was in progress. Another question dealt with whether personnel thought the incident could escalate.

For peer review only - http://bmjopen.bmj.com/site/about/guidelines.xhtml

Other questions related to situation awareness and general chemical preparedness. For example, one question dealt with which information personnel would need to be certain of whether a person's symptoms could be related to a chemical incident. Relevant follow-up questions were asked. A test interview was performed that enabled the development of the interview guide. All interviews were performed via Zoom or Teams. The interviews were performed between February and September 2021. The interviews ranged from 34 minutes to 72 minutes, with a mean of 63 minutes. All interviews were recorded and transcribed verbatim.

Data analysis

The individual interviews were analysed using qualitative content analysis (19-21). The transcripts were first read in order to gain an overall understanding of the content. Meaning units relating to the aim were extracted from the text and used to create codes. The codes were sorted into categories and sub-categories. Relevant citations were used to illustrate the codes and categories. The first author performed the coding and categorisation. The categorisation was then discussed with the second author and iteratively improved. A summary in the beginning of the results has also been added to describe the general situation awareness of the ERC operators. This was done since much of what the ERC personnel say they do in all emergency calls will also influence their work during chemical incidents.

Ethical considerations

This study was performed in accordance with the Helsinki Declaration (22). Participants were informed of the study objectives, both verbally and in writing, and were guaranteed confidentiality and the right to withdraw from the study at any time. The participants gave their written informed consent to participate in the study. The study's objectives and methods

For peer review only

are not covered by the Swedish Act concerning the Ethical Review of Research Involving Humans (23).

RESULTS

Before going through the results relating to chemical incidents in more detail, a brief summary of the general situation awareness of the ERC personnel influencing their work during chemical incidents will be presented. The ERC operators mentioned that they usually quite quickly ascertain whether a given situation deviates from normal patterns. Operators can do this because of their experience of hearing details which others cannot hear, such as what is happening in the background of the call, or nuances in the phrasing and pitch of the callers. They described themselves as detectives who trust their gut feelings. If something felt wrong, personnel followed up with probing questions, and judged that they were correct in 95% of the cases. The ERC operators also asserted that they had to be able to adapt their style of interviewing depending on the caller - for example, by being empathetic or authoritative, but always being calm and professional. Difficult situations included those where the caller was not cooperative, where language barriers were present (despite access to interpreters), where the location was unclear, or when a chaotic situation prevailed at the incident site. In better situations, callers were cooperative, and could even be asked to provide an overview of the incident site, meet up with the rescue service and emergency medical services, or warn others not to enter the incident area.

This study resulted in three categories of responses (Table 1), centring on 1) the complexity of identifying chemical incidents, 2) the importance of ensuring the safety of citizens and personnel of emergency organizations, and 3) the situation-based dispatch of organizations.

For peer review only - http://bmjopen.bmj.com/site/about/guidelines.xhtml

Table 1 Categories and sub-categories of interview responses

Category	Sub-category
The complexity of identifying chemical incidents	Perceiving the chemical incident and its severity
	Identifying chemical substances based on the situation
The importance of ensuring the safety of citizens and personnel of emergency organizations	Giving advice to the caller about safety
	Considering the safety of the emergency organizations' personnel
The situation-based dispatch of organizations	Balancing the need for units with their availability
	Considering which other organizations to connect to the emergency call

For peer review only

The complexity of identifying chemical incidents

The ERC personnel assert that it is often difficult to gain situation awareness about whether a chemical incident is actually in progress, what kind of chemicals are involved, how dangerous the situation is, and its potential consequences.

Perceiving the chemical incident and its severity

Chemical incidents were considered as rare but potentially severe events, varying from singular chemically injured persons to major chemical incidents. The ERC personnel considered major chemical incidents as likely, and recognised that they have to be prepared for them. They considered themselves reasonably well prepared, and asserted that chemical incidents were not more difficult to manage than other incidents. However, participants stated that they became more focused and had to think outside the box when such incidents occurred, especially if they are unsure about the chemical reactions taking place, or in particularly difficult situations such as fires or incidents at sea.

For peer review only - http://bmjopen.bmj.com/site/about/guidelines.xhtml

Because of the rarity of chemical incidents, the ERC personnel stated that there was a risk that they would not perceive that a given incident indeed was chemical in nature. Thus, in order to not dismiss a dangerous situation, participants mentioned that they spend more time on incidents they perceived as vague than on obvious ones. Participants mentioned that they have certain trigger words for chemical incidents, which were emission, leakage, traffic incident, strong strange smell, if people are feeling sick without a clear cause, smoke or gas leaks, dripping sounds, that something looks strange, fires in industries or cars, or whether a heavy goods vehicle is involved in the incident.

Chemical incidents were also considered as vague incidents because the chemicals can be invisible, which makes it difficult to assess the volume of the release. Participants also mentioned the difficulties of understanding the chemical incident's potential to escalate and become more dangerous in a short time. For example, incidents can escalate if a fire starts or spreads, if there is a combination of cargo, if the chemical substance comes into contact with water, or if the incident has occurred on a heavily trafficked road.

Another kind of difficult situation entailed having to identify symptoms (such as affected breathing, cough, stinging eyes, nausea, headache and dizziness) as being caused by a chemical incident, because these are such common symptoms. Other ERC personnel considered that chemical incidents could be obvious - for example incidents with a heavy goods vehicle transporting dangerous chemicals. The ERC personnel considered that it depended on the situation if they clearly comprehended that it was a chemical incident or not - some callers may be very verbal and factual regarding relevant information, while others may be less communicative when anxious:

> *They called and said that a person had inhaled chlorine gas at an ice-hockey stadium, which is not strange, it can happen. It never emerged in the call that it was still leaking. The callers never mentioned this, they were mostly shocked.*

For peer review only - http://bmjopen.bmj.com/site/about/guidelines.xhtml

Then the ERC operator had to ask "is it still leaking?". It took about 20-30 seconds to ask that question, because it didn´t emerge spontaneously. (Rescue service dispatcher)

Identifying chemical substances based on the situation

ERC personnel asserted that it was important to quickly identify the chemical substance or substances in play, as well as how dangerous it or they were, but also felt that this was not always easy. One ERC operator mentioned that the caller is their eyes on site. In order to assess the situation and find out which chemical is involved, there are a lot of questions the ERC operators could ask - for example, what does the smoke/substance look like, how does it smell or if observers can see any orange warning signs at heavy goods vehicles. The ERC operators acknowledged that they could be ignorant about chemicals and their effects, and could be in need of more information from the rescue service, the specialist emergency medical service for chemical incidents or the Swedish Poisons Information Centre. The rescue service dispatchers mentioned that they were knowledgeable about chemicals and could also search for more information in the chemical substance database.

If we receive a chemical formula that we don't recognise, we search the chemical substance database in order to be able to notify the rescue service when they are on their way. We get to know that the chemical substance can react with oxygen and can be ignited at 27°C and it is 22°C outside. This means lives are at risk for those who are at the site of the incident. (ERC operator)

The ERC personnel felt that the general public had minimal knowledge about chemicals and thus preferred if professional personnel (e.g. heavy goods vehicle drivers and industry workers) made the emergency call. The professionals are trained in what to say - they know which chemical substances they handle, and are knowledgeable about which risks a given

For peer review only - http://bmjopen.bmj.com/site/about/guidelines.xhtml

substance entails. In certain cases, e.g. if a driver is injured, the ERC personnel could call the transportation business to ask what the heavy goods vehicle is transporting. However, during major incidents, e.g. major industry incidents, the ERC operators stated that it could be really difficult to gain information from the professional personnel. They mentioned that their first operational picture could change really quickly while the call progressed and they received more information.

The importance of ensuring the safety of citizens and personnel of emergency organizations

The ERC personnel stated that they have to attain situation awareness about the chemical incident in order to ensure the safety of both the citizens at the incident site and the emergency organization personnel that will arrive at the incident site.

Giving advice to the caller about safety

The ERC operators considered it difficult to give advice to callers during chemical incidents, but acknowledged that they had enough experience and common sense to give sound advice. Most of the time, operator advice was generic, but operators could also give advice from a chemical substance database or based on recommendations from the rescue service. Examples of advice were to move further away from the incident and be mindful of the direction of the wind, to evacuate buildings and wait for the rescue service to rescue trapped persons.

While the ERC operators volunteered that they want to receive as much information as possible, they still saw their prime responsibility as ensuring the safety of the caller. Thus, they have to be careful when asking callers to investigate the scene - for example, in case of missed warning signs. If there are others injured at the scene of the incident, they are a secondary priority if there is a risk of even more casualties. Operators usually had to be very

For peer review only - http://bmjopen.bmj.com/site/about/guidelines.xhtml

straightforward with the callers in telling them to think of their own safety, leave injured persons behind and advise them against running into dangerous situations, as well as in advising callers to keep distance while waiting for experts with appropriate equipment. Sometimes the caller wants to help but does not see the seriousness of the situation, and this might lead to an even worse situation, despite their good intentions.

> *When it comes to animals which may burn to death inside a barn or when there are still people left in a burning building, those scenarios are difficult, because people want to run in, most of them anyway. We want them to keep as far away as possible in order for them not to hurt themselves. (ERC operator)*

Considering the safety of the emergency organizations' personnel

The ERC personnel considered it important that they correctly inform the emergency organizations of the chemical incident. Despite not always knowing the chemical substance involved, participants verify that the emergency organizations perceive that it is a dangerous situation. Thus, ERC personnel would rather give a bit too much information than too little, so that the emergency organizations can make their own assessments of the situation. If ERC personnel have knowledge of the chemical substance involved, they can give relevant safety advice, for example on safety distances. When they do not know the chemical substance involved, the dispatchers considered it to be difficult to indicate to the emergency organizations that a chemical incident actually is in progress. If they do not know the chemical substance(s) involved, or have very little information about the incident, ERC personnel report that they will work as if the worst possible situation prevailed, and then (if called for) de-escalate the situation, based on information from the emergency organizations at the incident site.

For peer review only - http://bmjopen.bmj.com/site/about/guidelines.xhtml

Interviewer: If you had received information that it was hydrochloric acid, what decisions would you then have made?

Rescue service dispatcher: I probably would not really have made any other decisions. However, I could have planned the rescue operation and what resources were necessary. I probably would not have needed as many resources, they could have extinguished the fire earlier at a closer range.

The situation-based dispatch of organizations

During chemical incidents, the ERC personnel need situation awareness in order to interpret the situation, as well as to inform and dispatch the required organizations. Participants believed that more resources are needed during chemical incidents than during other incidents, but also emphasise that limits on resources must also be taken into consideration.

Balancing the need for units with their availability

The dispatchers decide which emergency organization units should be dispatched to an incident based on the first incoming information from the callers, pre-made plans or directives, and the competences of the dispatched units (e.g., the specialist emergency medical service for chemical incidents). This means they have to dare to make quick decisions about how many units they should dispatch to the incident.

The rescue service would rather dispatch 10 times too many units than miss dispatching units when we could have saved someone. We are drilled that if we happen to dispatch two rescue service units too many, we can just withdraw them if they are not needed. (Rescue service dispatcher)

The dispatchers' decisions about the number of dispatched units differ depending on the situation - for example, the risk for further injuries, quantity of chemical substances involved,

For peer review only - http://bmjopen.bmj.com/site/about/guidelines.xhtml

and type of environment. Participants assert that chemical incidents demand more units than other incidents - something which often requires that extra emergency medical service units be sent, because there is a risk of injury to rescue service personnel. If a major incident is in progress, dispatchers will send all the available units in the region. However, the emergency medical service dispatchers considered they had to prioritise where they send their units, because they also have a responsibility to care for all other patients with severe injuries and illnesses. For example, in a city they may be able to send all available units to a chemical incident, but not in the countryside because of long distances.

The dispatchers report working with the resources they have available - for example, moving resources in the region to strategic places in order to cover the entire region. They can also collaborate with other regions in order to increase the available resources. The dispatchers will also have a dialogue with the emergency organizations at the incident site if more resources are needed and they can give suggestions of additional available resources. It is the incident commanders who decide which resources are needed based on the situation at the incident site, in relation to the available resources.

Considering which other organizations to connect to the emergency call

The ERC operators report that they quickly have to consider, contact and inform several organizations and chemical experts, apart from the rescue service and emergency medical dispatchers. For example, they report that there are specialised rescue services which are contacted if an incident happens within their area of responsibility, such as the sea, mountain, industry or airport rescue service. The ERC operators also described that they contact, ask for more information and inform the police response centre operators that dispatch police units to the chemical incident.

When I understand that it's an incident with several [people] exposed to the same substance, I contact the rescue service and the police. I ask them if they know anything and give them the information we have, that there are several persons that have been exposed and we suspect that it's this substance but we have no idea, but there are people that have been in a specific place and they start to show symptoms and something isn't right. (ERC operator)

Other relevant representatives the ERC personnel mentioned that they can consider contacting, including officials on call, the salvage officer and representatives from the Swedish Transport Administration. The officials on call are contacted for resource allocation and preparation of the hospitals in the region. The salvage officer works together with the rescue service to protect the environment - for example, by taking care of spilled chemicals and by documenting the incident. Representatives from the Swedish Transport Administration are contacted if the ERC personnel consider it important to block a road or reroute traffic. If there is a major chemical incident, they also contact the Swedish Civil Contingencies Agency, who inform the citizens of the chemical incident through an Important Public Announcement. This could for example happen if there is a risk to the health and safety of the citizens due to toxic smoke, or if an area is blocked off due to the risk of explosion.

For peer review only

DISCUSSION

This study aimed at examining the situation awareness of the personnel working at Emergency Response Centres - how they perceive, comprehend, project and act during chemical incidents. Responses could be sorted into three categories: the complexity of identifying chemical incidents, the importance of ensuring the safety of citizens and personnel of emergency organizations, and the situation-based dispatch of organizations. These results are discussed further below.

For peer review only - http://bmjopen.bmj.com/site/about/guidelines.xhtml

Starting with the complexity of identifying chemical incidents, in this study the ERC personnel reported that it could take time to ascertain whether a situation was dangerous or had the potential to escalate. Prior research has also confirmed that ERC personnel can receive inadequate information or have difficulties in grasping the situation (24). During chemical incidents this can be especially true if the incident type is not covered by personnel interview guides, or if the information about the incident is fragmented and several callers have to provide information for a full understanding of the situation, as reported in another study (25).

Prior literature also indicates that it is especially important to be vigilant during motor vehicle collisions, heavy goods vehicle incidents or structural fires, because for example the hazmat placard could have been damaged in the heavy goods vehicle crash (3). The ERC personnel interviewed in this study stated that hazard plaques and environmental clues could be of great help, but they also gave examples of when they contacted the owner of the heavy goods vehicles involved if they were uncertain of the chemical substance(s) at play, something which may need to be done if the involved driver is unable to answer any questions (3). A suggestion to further improve the knowledge of the ERC personnel on chemical incidents and implement lessons learned could be to develop emergency organization use of chemical incident databases, similarly to what is suggested in the chemical industry literature (26).

Another topic that was discussed in this study was that interviewed ERC personnel emphasised how important it could be that professional personnel made the emergency call, since other professionals often are easier to talk to than other callers. Generally, the ERC personnel interviewed in other studies also state that it is more difficult to understand information and assess the severity of the symptoms from third-party callers, such as relatives or strangers at an incident site, who do not have any background information about any sick or injured persons (27). Thus, improving the public knowledge and ability to act during

For peer review only - http://bmjopen.bmj.com/site/about/guidelines.xhtml

chemical incidents by performing pre-incident information campaigns about chemical incidents (28) could possibly lead to ERC personnel gaining more useful information from the public.

The importance of ensuring the safety of citizens and personnel of emergency organizations is a category of responses emphasising the importance of ERC personnel's situation awareness for their ability to give correct information and sound advice, because the chemical incident site may still be dangerous. Prior literature also states that it is important for ERC personnel to be able to assess risks, reassure and give the caller appropriate instructions and safety advice, as well as to gather and share information with the emergency organizations in order to prepare them - both tasks, when executed, will decrease the risks to all involved (16, 24, 25, 29). This is important because it is relatively common that the personnel of the emergency organizations are injured during chemical incidents (30). Thus, the ERC personnel of this study stated that it was important to inform the emergency organizations about the situation at the incident site, including information about the chemical substance if they knew it.

Further, prior literature has also pointed out that if the chemical substance in play is known, so is the route of exposure and the likelihood for secondary contamination of the emergency organization personnel, as well as the most effective method of protection and decontamination (3). Further, stressing the importance of correct information from the ERC personnel, another study (31) states that there is a risk that the emergency organizations are misguided, because they form their impressions of the incident based on pre-arrival information rather that post-arrival information. This is also discussed in another study, which state that incomplete information from the ERC personnel could lead the emergency medical service units to misinterpret the situation and lead to wrong actions, which in turn leads to delayed care of those affected (32). Thus, the importance of the ERC personnel to ensure the safety of both citizens and emergency personnel cannot be stressed too much.

For peer review only - http://bmjopen.bmj.com/site/about/guidelines.xhtml

Another study has found that so-called reflection seminars can be helpful in focusing on unsolved problems in structures, behaviours and working methods, as well as in confirming existing knowledge and procedures (33). Such seminars may also build trust and collaboration between emergency organizations, and complement learning obtained from full-scale exercises (33). Thus, in order to decrease the risk of injuries to emergency personnel and citizens, it could potentially be of value to create forums for all responding organizations including the ERC personnel, where learning from each other about safety during potential chemical incidents could be discussed. Lessons learned could then be further inculcated and put into practice during full-scale exercises.

Finally, the importance of the situation-based dispatch of organizations was in this study confirmed by ERC personnel reports that they usually worked with a given understanding of which organizations and units needed to be informed and dispatched to the chemical incident. However, although the ERC personnel in this study stated that chemical incidents demanded a lot of units, they also had to take into consideration how many units they actually had available and prioritise between patients. This is consistent with what other authors state, that ERC personnel have to consider that there are limited resources and that they had to prioritise the calls in relation to other waiting calls (12, 13). Thus, also prior literature states that ERC personnel have to be aware of what is happening in the area regarding available resources and other ongoing incidents in order to send sufficient resources (7).

Another issue relating to the issues of limited resources is that chemical incidents can be perceived as vague, as shown in this study's results. One prior study has also found that sending resources to vague but urgent alarms could be problematic if the situation responded to was found to be non-urgent upon arrival (29). Thus, ERC personnel have to balance the need of resources with the availability of resources. Reflection seminars could provide a

For peer review only - http://bmjopen.bmj.com/site/about/guidelines.xhtml

forum where ERC personnel can discuss resource management during chemical incidents with representatives from the emergency organizations.

Strengths and limitations

One strength of the study is that the participants come from different ERCs - thus, we can gain a geographically diverse understanding of the situation awareness of ERC personnel. One limitation is that the Swedish system is very much a diverse system when it comes to the roles and responsibilities and even education of the Emergency Response operators and emergency organizations' dispatchers. This may lead to a fragmented understanding of personnel's perspectives - perhaps, a greater number of interviews would have been necessary to cover more aspects of the study. The interviews were also performed via Zoom and Teams due to Covid-19, which can influence the quality of the interviews. The participants themselves could choose if they wanted to have a videocall or if they did not want their picture showing, in order for them to feel as comfortable as possible. However, all in all, the participants gave a varied understanding of how it is to work during chemical incidents and the interviews were rich and in general about one hour in length, which resulted in a lot of material to analyse.

Practical implications

From this study we can conclude that the situation awareness of the ERC personnel is vital in order for them, the citizens at the incident site and the personnel of the emergency organizations to manage the chemical incident in a safe and correct way. In order to further increase the situation awareness of ERC personnel, a practical implication of this study could be that there is a need to increase citizens' knowledge on chemical incidents, so that it is more likely that they give relevant information to ERC personnel. This could be an area where more research and development are needed.

For peer review only - http://bmjopen.bmj.com/site/about/guidelines.xhtml

Further, in order to increase the knowledge of chemical incidents among both ERC personnel and the personnel of emergency organizations, a chemical incident database could be added to the chemical substance database. In this database, all incidents could be documented together with their responses and lessons learned. These and other safety and dispatch-related questions could then be continually discussed during reflection seminars for ERC and emergency organization personnel, so that experiences can be shared. This learning could then be further improved during joint training and full-scale exercises. All exercises could also include elements found in this study to train the situation awareness of the participants.

CONCLUSION

This study highlights the necessity for ERC personnel to quickly attain accurate situation awareness of what is happening during potentially dangerous chemical incidents in order to be able to notify, inform and dispatch the correct resources. During chemical incidents there is a dichotomy between the ERC personnel wanting as much information as possible for everyone's safety, also for emergency organizations, and their responsibility for the safety of the caller. This is not always a straightforward task, especially since chemical incidents can be considered vague. This study suggests that in order to increase the situation awareness of ERC personnel during chemical incidents, interventions to increase public knowledge of chemical incidents could be performed, chemical incident databases could be added to the chemical substance database, and reflection seminars complementing full-scale exercises could be utilised to investigate problems. These areas could also be interesting to study further.

Acknowledgement The authors would like to thank all participants for their contribution to the research. The authors would also like to thank professor Britt-Inger Saveman for helpful suggestions during the conception of the study and interview guide and recruitment of

For peer review only - http://bmjopen.bmj.com/site/about/guidelines.xhtml

participants. The authors would also like to thank Raimo Lindgren for help with performing four of the interviews. We would also like to thank Johanna Björnstig for writing the transcriptions of the interviews.

Contributors SK, LG and BIS conceived and designed the study. SK interviewed all but four of the participants, which was performed by RL. SK performed the first analysis and wrote the first draft of the manuscript both which was concurrently discussed with LG and revised. The final manuscript is approved by both SK and LG. LG is the guarantor.

Patient and public involvement in research Patients or the public were not involved in the design, conduct, reporting or dissemination of this research.

Ethics approval The study's objectives and methods are not covered by the Swedish Act concerning the Ethical Review of Research Involving Humans. Thus, approval from the Swedish Ethical Review Authority for this study has not been sought.

Competing interests None declared

Funding The research was funded by the Swedish National Board of Health and Welfare, grant number N/A.

Data availability statement All data relevant to the study are included in the article or uploaded as supplementary information.

Patient consent for publication Not applicable

REFERENCES

1. World Health Organization. Manual for the public health management of chemical incidents. Geneva:2009.

For peer review only - http://bmjopen.bmj.com/site/about/guidelines.xhtml

2. Newmark J. Clinical aspects of large-scale chemical events. In: Koenig KL, Schultz CH, eds. Koenig and Schultz's disaster medicine comprehensive principles and practice. 2nd edition. Cambridge, Cambridge University Press: 2016:499–521.

3. Lim HC, Tan HH. Hazardous material, toxic, and industrial events. In: Koenig KL, Schultz CH, eds. Koenig and Schultz's disaster medicine comprehensive principles and practice. 2nd edition. Cambridge, Cambridge University Press: 2016:579–599.

4. United Nations Office for the Coordination of Humanitarian Affairs (OCHA). Lebanon: Beirut Port Explosions Situation Report No.1 (As of 5 August 2020) [online]. 2020. https://reliefweb.int/report/lebanon/lebanon-beirut-port-explosions-situation-report-no-1-5-august-2020-enar (accessed 29 November 22).

5. TT. Ammoniakutsläpp i Helsingborg (Swe). Ammonia emissions in Helsingborg. [online] 2018. from https://www.svt.se/nyheter/inrikes/ammoniakutslapp-i-helsingborg. (accessed 29 November 2022).

6. Lindberg J. Brand på Northvolt: Flera personer till sjukhus (Swe) [online]. Fire at Northvolt: Several people taken to hospital. Västerbottenskuriren. 2022. https://www.vk.se/2022-10-07/brand-pa-northvolt-flera-personer-till-sjukhus (accessed 28 November 2022).

7. Norri-Sederholm T, Seppälä J, Saranto K, et al. Information flow and situational awareness in emergency medical dispatch. International Journal of Networking and Virtual Organisations. 2016;16(1):72–85. doi: https://doi.org/10.1504/IJNVO.2016.075134

8. Swedish Government Official Reports. SOU 2018:28 En nationell alarmeringstjänst - för snabba säkra och effektiva hjälpinsatser (Swe). SOU 2018:28 A national emergency response center - for rapid, secure and effective rescue operations. Ministry of Justice, Stockholm:2018.

For peer review only

9. Magnussen LI, Carlstrøm E, Berge AK, et al. Help we are sinking! Stories from Norwegian dispatch centers on decision-making in unfamiliar and ambiguous situations. Journal of Emergency Management. 2018;16(4):245–254. doi: https://doi.org/10.5055/jem.2018.0373

10. Blandford A, Wong BLW. Situation awareness in emergency medical dispatch. International Journal of Human-Computer Studies 2004;61(4):421–452. doi: https://doi.org/10.1016/j.ijhcs.2003.12.012

11. Endsley MR. Toward a theory of situation awareness in dynamic systems. Human Factors: The Journal of the Human Factors and Ergonomics Society 1995;37(1):32–64. doi: https://doi.org/10.1518/001872095779049543

12. Leonardsen A-C, Ramsdal H, Olasveengen TM, et al. Exploring individual and work organizational peculiarities of working in emergency medical communication centers in Norway – a qualitative study. BMC Health Services Research. 2019;19:545 https://doi.org/10.1186/s12913-019-4370-0

13. Ek B, Svedlund M. Registered nurses' experiences of their decision-making at an Emergency Medical Dispatch Centre. Journal of Clinical Nursing. 2015;24(7-8):1122–1131, doi: https://doi.org/10.1111/jocn.12701

14. Torlén Wennlund K, Kurland L, Olanders K, et al. Emergency medical dispatchers' experiences of managing emergency calls: a qualitative interview study. BMJ Open. 2022;12:e059803. doi: 10.1136/bmjopen-2021-059803

15. Rispens JR, Jones SA, Clemmons NS, et al. Anhydrous ammonia chemical release - Lake County, Illinois, April 2019. Morbidity and Mortality Weekly Report. 2020;69(4):109–113. doi: 10.15585/mmwr.mm6904a4

For peer review only - http://bmjopen.bmj.com/site/about/guidelines.xhtml

16. Gardett I, Scott G, Broadbent M, et al. Situational awareness in emergency medical dispatch: An obervation study and proposed model. Annals of Emergency Dispatch and Response 2019;7(2):16–22.

17. The National Board of Health and Welfare in Sweden. Händelser med kemikalier - Kunskapsunderlag för hälso- och sjukvården (Swe). Chemical incidents - Document of knowledge for the health care. Stockholm: 2009.

18. Uppsala University Hospital. Sjukvårdens larmcentral (Swe). Emergency medical dispatch center [online]. 2020. https://www.akademiska.se/for-vardgivare/verksamhetsomraden/ambulanssjukvard/larmcentral/ (accessed 28 November 2022).

19. Graneheim UH, Lundman B. Qualitative content analysis in nursing research: concepts, procedures and measures to achieve trustworthiness. Nurse Education Today 2004;24(2):105–112. doi: https://doi.org/10.1016/j.nedt.2003.10.001

20. Graneheim UH, Lindgren B-M, Lundman B. Methodological challenges in qualitative content analysis: A discussion paper. Nurse Education Today 2017;56:29–34. doi: https://doi.org/10.1016/j.nedt.2017.06.002

21. Lindgren B-M, Lundman B, Graneheim UH. Abstraction and interpretation during the qualitative content analysis process. International Journal of Nursing Studies. 2020;108:103632. doi: https://doi.org/10.1016/j.ijnurstu.2020.103632

22. World Medical Association. World Medical Association Declaration of Helsinki - Ethical principles for medical research involving human subjects [online]. 2013. from https://www.wma.net/policies-post/wma-declaration-of-helsinki-ethical-principles-for-medical-research-involving-human-subjects/ (accessed 29 November 2022).

For peer review only

23. Swedish Code of Statutes. Lag (SFS 2003:460) om etikprövning av forskning som avser människor (Swe). Law concerning the ethical review of research involving humans. The ministry of education and research. Stockholm:2003.

24. Forslund K, Kihlgren A, Kihlgren M. Operators' experiences of emeregency calls. Journal of Telemedicine and Telecare 2004;10(5):290–297. doi: https://doi.org/10.1258/1357633042026323

25. Westman A, Saveman B-I, Björnstig U, et al. Mobilisation of emergency services for chemical incidents in Sweden - a multi-agency focus group study. Scandinavian Journal of Trauma, Resuscitation and Emergency Medicine. 2021;29:99. doi: https://doi.org/10.1186/s13049-021-00910-5

26. Wood MH, Koutelos K, Hailwood M, et al. Learning lessons from chemical incidents – What's stopping us and how we can make it happen. Chemical Engineering Transactions 2022;90:685–690. doi: https://doi.org/10.3303/CET2290115

27. Holmström IK, Kaminsky E, Lindberg Y, et al. The perspectives of Swedish registred nurses about managing difficult calls to emergency medical dispatch centres: a qualitative descriptive study. BMC Nursing 2021;20:150. doi: https://doi.org/10.1186/s12912-021-00657-5

28. Carter H, Weston D, Symons C et al. Public perceptions of pre-incident information campaign materials for the initial response to a chemical incident. Disaster Prevention and Management 2019;28(5):565–584. doi: https://doi.org/10.1108/DPM-10-2018-0342

29. Palsgaard Møller T, Gamst Jensen H, Viereck S, et al. Medical dispatchers' perception of the interaction with the caller during emergency calls – a qualitative study. Scandinavian Journal of Trauma, Resuscitation and Emergency Medicine. 2021;29:45 doi: https://doi.org/10.1186/s13049-021-00860-y

For peer review only - http://bmjopen.bmj.com/site/about/guidelines.xhtml

30. Melnikova N, Wu J, Yang A, et al. Acute chemical incidents with injured first responders, 2002-2012. Disaster Medicine and Public Health Preparedness. 2018;12(2):211–221. doi: https://doi.org/10.1017/dmp.2017.50

31. Keene T, Pammer K, Lord B, et al. Dispatch information affects diagnosis in paramedics: an experimental study of applied dual-process theory. International Journal of Emergency Services 2022;11(2):277–291. doi: https://doi.org/10.1108/IJES-06-2021-0039

32. Hernborg MN, Carlström ED, Berlin JM. First initiatives in prehospital care – Basing assessments on incomplete preliminary information. Journal of Nursing Education and Practice 2020;10(6):47–54. doi: https://doi.org/10.5430/jnep.v10n6p47

33. Roud E, Gausdal AH, Asgary A, et al. Outcome of collaborative emergency exercises: Differences between full-scale and table-top exercises. Journal of Contingencies and Crisis Management 2021;29(2):170–184. doi: https://doi.org/10.1111/1468-5973.12339

For peer review only

For peer review only - http://bmjopen.bmj.com/site/about/guidelines.xhtml

Interview guide: Situation awareness, Emergency Response Centre personnel

General information about the participant:

- How old are you?
- How many years have you worked at the Emergency Response Centre?
- Are you an operator/rescue service dispatcher/emergency medical service dispatcher?
- Have you previously worked with something that can be relevant considering that the interview is about chemical incidents? If yes – for how many years?
- What kind of education do you have?

Interview questions:

1. Can you describe a situation where you received an emergency call regarding an incident, immediately or later classified as a chemical incident, where there was a risk of people being injured or dying?
    a. What made you understand that it was more than an ordinary incident – an incident with chemicals involved when there was a risk of casualties?
        i. Was it something the caller said?
        ii. How did you find out the extent of the incident, for example if there were several people injured?
        iii. Was it evident from the start that it was a chemical incident with a risk for casualties?
    b. When you understood that it was a chemical incident with a risk for casualties, did you have access to an interview guide or other guide?
        i. What made you choose a specific interview guide?
            1. Do you ever need ask other questions than those in the interview guide? If yes – why?
            2. If you needed to ask other questions than those in the interview guide, whom did you ask and what did you ask?
        ii. Did you receive information about which chemical substance was involved?
            1. How do you generally receive information about which chemical substance is involved? / How do you usually do to receive information about which chemical substance is involved?
            2. Depending on the chemical substance, how did you have to adjust your questions in order to know how you should act?
    c. Did you consider that the incident could become dangerous for more people? /escalate?
        i. Why? / why not?
        ii. How did you think the situation could develop?
            1. Before the emergency organizations arrived?
                a. Do you manage the emergency calls about chemical incidents differently depending on the lead time, e.g., if

For peer review only

the incident is in a sparsely populated area or quite close by?

2. When the emergency organizations were at the incident site?
3. When the emergency organizations had left the incident site? (Is there a continued risk?)
    a. During major chemical incidents, the incident site can be polluted/unsafe for a long time - do you have continual preparedness for several people being injured at the same incident site?

d. What decisions did you make?
    i. What information did you give to the caller, what should he/she do?
        1. What do you think of the dilemma of safety versus saving lives?
    ii. How did you know which resources needed to be dispatched?
        1. Are there dispatch plans?
    iii. Is there a particular order in which you contact the emergency organizations? If yes – does this differ depending on if it is a chemical incident with the risk for casualties?
    iv. How do you inform the emergency organizations that it is a chemical incident with the risk for casualties?
    v. Which information do you expect the emergency organizations at the incident site to send to you?
    vi. How does the information you receive from the emergency organizations at the incident site influence your actions?
    vii. Do the decisions you take differ depending on the chemical substance involved?
    viii. Do your decisions differ depending on if you suspect an antagonistic incident and not a regular incident?

General questions to ask after the participants have described their experiences

1. Is there anything regarding emergency calls about chemical incidents with the risk for casualties that you consider challenging? - What? Why? / Why not?
    a. Do you need the support of a back office/ other support during chemical incidents? Do you sit together?
    b. Are there occasions when you feel that the incident is worse than what the caller thinks? – In that case, what do you do?
2. Are there situations when you more often consider that it can be a chemical incident with the risk for casualties?
    a. If an alarm comes from a Seveso facility, for example, does this influence how you think about the incident?
        i. Is there any extra information that you would ask for?
    b. Depending on if it is a citizen that makes the emergency call, a professional working at a Seveso facility, or a heavy goods vehicle driver, do you think differently?
    c. If an electric bus start burning, how do you proceed?
        i. Are there special tasks you need to perform?
        ii. How do you think the situation could develop if this happened in a tunnel/residential area?

For peer review only

3. If you receive an emergency call that suggests that the person's symptoms could be a consequence of a chemical incident, which information would you need to be certain?
    a. If several emergency response centre operators receive calls about persons with the same symptoms, how would you understand if it was the same chemical incident?
    b. During chemical incidents the symptoms does not always present at once. Do you usually take this into consideration when you make decisions?
4. How do you work with the operator/dispatchers during joint emergency calls during chemical incidents with the risk for casualties? Is there any difference compared to other major incidents?
    a. How do you ensure that all persons in the joint emergency call have the same information about the chemical incident?
5. What responsibility do you have when you have dispatched the emergency organization units and they have arrived at the incident site?
    a. Do you need to be prepared to adjust the emergency organization units depending on how serious the incident is and how many injured there are?
    b. If, during a chemical incident, people in the emergency organization units would become injured due to lacking information, what would you think of such an incident?
6. Is there anything which we have not talked about that you want to bring up? Do you have any questions?

For peer review only

COREQ (COnsolidated criteria for REporting Qualitative research) Checklist

A checklist of items that should be included in reports of qualitative research. You must report the page number in your manuscript where you consider each of the items listed in this checklist. If you have not included this information, either revise your manuscript accordingly before submitting or note N/A.

Topic	Item No.	Guide Questions/Description	Reported on Page No.
**Domain 1: Research team and reflexivity**			
Personal characteristics			
Interviewer/facilitator	1	Which author/s conducted the interview or focus group?	
Credentials	2	What were the researcher's credentials? E.g. PhD, MD	
Occupation	3	What was their occupation at the time of the study?	
Gender	4	Was the researcher male or female?	
Experience and training	5	What experience or training did the researcher have?	
Relationship with participants			
Relationship established	6	Was a relationship established prior to study commencement?	
Participant knowledge of the interviewer	7	What did the participants know about the researcher? e.g. personal goals, reasons for doing the research	
Interviewer characteristics	8	What characteristics were reported about the inter viewer/facilitator? e.g. Bias, assumptions, reasons and interests in the research topic	
**Domain 2: Study design**			
Theoretical framework			
Methodological orientation and Theory	9	What methodological orientation was stated to underpin the study? e.g. grounded theory, discourse analysis, ethnography, phenomenology, content analysis	
Participant selection			
Sampling	10	How were participants selected? e.g. purposive, convenience, consecutive, snowball	
Method of approach	11	How were participants approached? e.g. face-to-face, telephone, mail, email	
Sample size	12	How many participants were in the study?	
Non-participation	13	How many people refused to participate or dropped out? Reasons?	
Setting			
Setting of data collection	14	Where was the data collected? e.g. home, clinic, workplace	
Presence of non-participants	15	Was anyone else present besides the participants and researchers?	
Description of sample	16	What are the important characteristics of the sample? e.g. demographic data, date	
Data collection			
Interview guide	17	Were questions, prompts, guides provided by the authors? Was it pilot tested?	
Repeat interviews	18	Were repeat inter views carried out? If yes, how many?	
Audio/visual recording	19	Did the research use audio or visual recording to collect the data?	
Field notes	20	Were field notes made during and/or after the inter view or focus group?	
Duration	21	What was the duration of the inter views or focus group?	
Data saturation	22	Was data saturation discussed?	
Transcripts returned	23	Were transcripts returned to participants for comment and/or	

For peer review only

Topic	Item No.	Guide Questions/Description	Reported on Page No.
		correction?	
**Domain 3: analysis and findings**			
Data analysis			
Number of data coders	24	How many data coders coded the data?	
Description of the coding tree	25	Did authors provide a description of the coding tree?	
Derivation of themes	26	Were themes identified in advance or derived from the data?	
Software	27	What software, if applicable, was used to manage the data?	
Participant checking	28	Did participants provide feedback on the findings?	
Reporting			
Quotations presented	29	Were participant quotations presented to illustrate the themes/findings? Was each quotation identified? e.g. participant number	
Data and findings consistent	30	Was there consistency between the data presented and the findings?	
Clarity of major themes	31	Were major themes clearly presented in the findings?	
Clarity of minor themes	32	Is there a description of diverse cases or discussion of minor themes?	

Developed from: Tong A, Sainsbury P, Craig J. Consolidated criteria for reporting qualitative research (COREQ): a 32-item checklist for interviews and focus groups. *International Journal for Quality in Health Care*. 2007. Volume 19, Number 6: pp. 349 – 357

Once you have completed this checklist, please save a copy and upload it as part of your submission. DO NOT include this checklist as part of the main manuscript document. It must be uploaded as a separate file.

For peer review only

BMJ Open

Situation awareness of Emergency Response Centre personnel during chemical incidents – an interview study in a Swedish context

Journal:	*BMJ Open*
Manuscript ID	bmjopen-2022-071347.R1
Article Type:	Original research
Date Submitted by the Author:	28-Apr-2023
Complete List of Authors:	Karlsson, Sofia; Umeå Universitet Medicinska fakulteten, Department of Surgical and Perioperative Sciences, Surgery<br>Gyllencreutz, Lina; Umea University Department of Surgical and Perioperative Sciences, Surgery
**Primary Subject Heading**:	Emergency medicine
Secondary Subject Heading:	Qualitative research
Keywords:	ACCIDENT & EMERGENCY MEDICINE, Telemedicine < BIOTECHNOLOGY & BIOINFORMATICS, QUALITATIVE RESEARCH

SCHOLARONE™
Manuscripts

I, the Submitting Author has the right to grant and does grant on behalf of all authors of the Work (as defined in the below author licence), an exclusive licence and/or a non-exclusive licence for contributions from authors who are: i) UK Crown employees; ii) where BMJ has agreed a CC-BY licence shall apply, and/or iii) in accordance with the terms applicable for US Federal Government officers or employees acting as part of their official duties; on a worldwide, perpetual, irrevocable, royalty-free basis to BMJ Publishing Group Ltd ("BMJ") its licensees and where the relevant Journal is co-owned by BMJ to the co-owners of the Journal, to publish the Work in this journal and any other BMJ products and to exploit all rights, as set out in our licence.

The Submitting Author accepts and understands that any supply made under these terms is made by BMJ to the Submitting Author unless you are acting as an employee on behalf of your employer or a postgraduate student of an affiliated institution which is paying any applicable article publishing charge ("APC") for Open Access articles. Where the Submitting Author wishes to make the Work available on an Open Access basis (and intends to pay the relevant APC), the terms of reuse of such Open Access shall be governed by a Creative Commons licence – details of these licences and which Creative Commons licence will apply to this Work are set out in our licence referred to above.

Other than as permitted in any relevant BMJ Author's Self Archiving Policies, I confirm this Work has not been accepted for publication elsewhere, is not being considered for publication elsewhere and does not duplicate material already published. I confirm all authors consent to publication of this Work and authorise the granting of this licence.

For peer review only

Situation awareness of Emergency Response Centre personnel during chemical incidents – an interview study in a Swedish context

Karlsson, Sofia[a]; Gyllencreutz, Lina[a] *

a. Department of Surgical and Perioperative Sciences, Surgery, Umeå University, 901 87 Umeå, Sweden

*Corresponding author, lina.gyllencreutz@umu.se

Word count: 5486

Tables: 1

Figures: 0

ABSTRACT

Objectives If a chemical incident occurs, the Emergency Response Centre (ERC) personnel are the first that are notified. They need to quickly attain situation awareness, based on the information from the caller, in order to dispatch the correct emergency units. The aim of this study is to examine the situation awareness of the personnel working at ERCs - how they perceive, comprehend, project and act during chemical incidents.

Methods Semi-structured individual interviews with 12 participants from the Swedish ERCs were performed. The interviews were analysed with qualitative content analysis.

Results Three categories of responses were identified. Responses focused on the complexity of identifying chemical incidents, the importance of ensuring the safety of citizens and personnel of emergency organizations, and the situation-based dispatch of organizations.

For peer review only

For peer review only - http://bmjopen.bmj.com/site/about/guidelines.xhtml

Conclusions The correct identification of the chemical incident and the involved chemical by the ERC personnel are necessary in order to notify, inform and dispatch the correct units, as well as to ensure the safety of citizens and emergency personnel. More research is needed about the dichotomies of the ERC personnel needing as much information as possible for everyone's safety versus their responsibility for the safety of the caller as well as between utilizing emergency dispatch index interview guides and trusting their gut feeling.

For peer review only

STRENGTHS AND LIMITATIONS OF THIS STUDY

- The combination of theory and experiences from ERC personnel on situational awareness in the event of a chemical incident strengthens the confirmability of this study.
- The geographical diversity of the participants in this study, as well as their differing professional roles strengthen the credibility of this study.
- The interview guide is transparent and therefore increases dependability, including consistency and repeatability of the study.
- A limitation of this study's transferability is that, due to a diverse system, the Swedish ERC personnel have different roles, responsibilities and education, depending on what ERC they operate at.

INTRODUCTION

Worldwide, large quantities of chemical substances are produced, transported and used, making incidents probable (1, 2). Incidents can result from human error or poor maintenance of equipment used for manufacturing and storage (3). Incidents can include fires, explosions, leaks and spills, and structural collapse (3). Chemical incidents involving the uncontrolled

For peer review only - http://bmjopen.bmj.com/site/about/guidelines.xhtml

release of toxic substances can potentially lead to injury or death of exposed persons, as well as harm the environment (1). A previous chemical incident that killed and injured many people was the Beirut port explosion in Lebanon on 4 August 2020 (4). However, most chemical incidents are small or medium-sized, and generally involve few killed or injured persons (1, 3), exemplified by a fire in a Swedish battery factory and an ammonia spill that injured about 10 persons respectively (5, 6).

Internationally as well as in Sweden, when an incident occurs, the first point of contact with the emergency organizations is often the Emergency Response Centres (ERC). The ERC operator is tasked with gaining as much relevant information from a caller as possible, and with determining which response is required (7). Dispatchers are connected to the call to inform and dispatch the correct emergency organization units (7-9). The ERC personnel swiftly manage a large quantity of information of varying quality from different sources, such as the general public or on-site emergency personnel, in order to make decisions in a dynamically changing situation (10). Thus, in order to do this, ERC personnel utilise their situation awareness, which means they perceive elements in the environment, comprehend the current situation and project its future status (11). The ERC personnel form a mental picture of the situation, where they use their experience and intuition in order to decide how to properly deal with a call (12). Because every call is unique, ERC personnel must be sensitive listeners, and should be able to reformulate their questions depending on the situation, balancing their use of the interview guide and their common sense and intuition (13, 14). However, an initial lack of information about the incident can result in injured first responders, as in the case of the release of anhydrous ammonia from a farm tractor towing a 2-ton ammonia tank experiencing mechanical failure in Illinois, USA (15). The ERC personnel initially reported the incident as a car fire, which led to several first responders being unaware of the danger. A number of responders were thus exposed to the gas, and some

For peer review only - http://bmjopen.bmj.com/site/about/guidelines.xhtml

later experienced symptoms of illness (15). Thus, the ERC personnel's situation awareness, related to their ability to identify the incident and predict medical outcomes for those affected, is highly important in chemical incidents.

The literature states that further study is needed on the challenges of situation awareness and behaviours used by the ERC personnel to overcome those challenges (16). Further, few studies focus solely on the ERC personnel's experiences of chemical incidents. Because of the prevailing danger during chemical incidents for the affected persons and responders alike, ERC personnel need to be able to manage the incoming information. This is especially true during major chemical incidents, when many persons may potentially be injured. Thus, the aim of this study is to examine the situation awareness of the personnel working at ERCs - how they perceive, comprehend, project and act during chemical incidents.

METHOD

Setting

In Sweden, ERC is an important part of the national emergency preparedness responding to approximate 3 000 000 calls a year. Depending on which ERC the personnel work at, they have different roles, responsibilities and education. The personnel at the ERC work either as an ERC operator, rescue service dispatchers or emergency medical service dispatcher. Some personnel are authorized to work in several positions, for example, variously working both as an ERC operator and a rescue service dispatcher.

The ERC operators are responsible to answer the national emergency number 112 and interview the callers based on index interview guide i.e. a criteria-based dispatch protocol structured in sub-indices. The dispatchers also listen to the call and can guide the operators to ask additional questions based on their specific expertise. The dispatchers should then dispatch and coordinate the emergency medical service and rescue service units. The ERC

For peer review only - http://bmjopen.bmj.com/site/about/guidelines.xhtml

operators are also collaborating with other organizations, such as the police (8, 17). In some Swedish regions, registered nurses perform the medical interview instead of an ERC operator (8, 18). The registered nurses uses more unrestricted guidelines with prompts based on caller descriptions of signs and symptoms, to provide direction and assistance in defining appropriate levels of care. In this study we have interviewed both operators, registered nurses and dispatchers.

Participants

Information about the study was provided in January 2021 to five managers of the ERCs located in various parts of Sweden. The managers notified their personnel of the study, and interested personnel with experience of chemical incidents were invited to participate. No time limit on when the incident occurred was considered necessary as the participants themselves chose the incident which they had experience of. In total, 12 respondents participated.

Nine of the participants were women and three were men. Their ages ranged from 32 to 63 years (with a mean age of 43), and their work experience at the ERCs varied from 2 to 32 years (with a mean length of eight years). Eleven participants worked as ERC operators. Of them five also worked as emergency medical service dispatchers whereof four were registered nurses. Additionally, five operators also worked as rescue service dispatchers. The last operator had exclusively that role. The twelfth participant exclusively worked as rescue service dispatcher.

Data collection

The semi-structured interview guide (see online supplemental file 1) was developed by the authors. The guide is inspired by the theory of situation awareness, applying the concepts of perception of elements in the current situation, comprehension of the current situation,

For peer review only - http://bmjopen.bmj.com/site/about/guidelines.xhtml

projection of future status and decision-making (11) to the ERC personnel's work during chemical incidents. In order to qualitatively capture the participants' own experiences in dealing with chemical incidents, the opening question was "Can you describe a situation where you received an emergency call regarding an incident, immediately or later classified as a chemical incident, where there was a risk of people being injured or dying". The interview questions then referred back to the described incident and situation awareness regarding that incident. The interview guide also contains questions that related to situation awareness and the participant's general chemical preparedness. A test interview was performed by the first author that enabled the development of the interview guide. All interviews were performed via Zoom or Teams. The interviews were performed between February and September 2021 by the first author and a research assistant. The interviews ranged from 34 minutes to 72 minutes, with a mean of 63 minutes. All interviews were recorded and transcribed verbatim by the first author and the research assistants.

Data analysis

The individual interviews were analysed with qualitative content analysis (19-21) using Microsoft Excel (2019) as management support. The transcripts were first read in order to gain an overall understanding of the content. Meaning units relating to the aim were extracted from the text and used to create codes. The codes were sorted into categories and sub-categories. Relevant citations were used to illustrate the codes and categories. The first author performed the coding and categorisation. The categorisation was then discussed with the second author and iteratively improved. A summary in the beginning of the results has also been added to describe the general situation awareness of the ERC operators. This was done since much of what the ERC personnel say they do in all emergency calls will also influence their work during chemical incidents.

For peer review only - http://bmjopen.bmj.com/site/about/guidelines.xhtml

Ethical considerations

This study was performed in accordance with the Helsinki Declaration and the Swedish Code of Statutes (22, 23). Participants were informed of the study objectives, both verbally and in writing, and were guaranteed confidentiality and the right to withdraw from the study at any time. The participants gave their written informed consent to participate in the study.

RESULTS

Before going through the results relating to chemical incidents in more detail, a brief summary of the general situation awareness of the ERC personnel influencing their work during chemical incidents will be presented. The general idea of what situational awareness is for the ERC personnel means in this result that they usually quite quickly ascertain whether a given situation deviates from normal patterns. Operators described that they can do this because of their experience of hearing details, such as what is happening in the background of the call, or nuances in the phrasing and pitch of the callers. They described themselves as detectives who trust their gut feelings and judged that their gut feeling was correct in most of the cases. If something felt wrong, personnel followed up with probing questions. The ERC operators also asserted the situational awareness to that they had to be able to adapt their style of interviewing depending on the caller - for example, by being empathetic or authoritative, but always being calm and professional.

This study resulted in three categories of responses (Table 1), centring on 1) the complexity of identifying chemical incidents, 2) the importance of ensuring the safety of citizens and personnel of emergency organizations, and 3) the situation-based dispatch of organizations.

Table 1 Categories and sub-categories of interview responses

**Category**	**Sub-category**

For peer review only - http://bmjopen.bmj.com/site/about/guidelines.xhtml

The complexity of identifying chemical incidents	Perceiving the chemical incident and its severity
	Identifying chemical substances based on the situation
The importance of ensuring the safety of citizens and personnel of emergency organizations	Giving advice to the caller about safety
	Considering the safety of the emergency organizations' personnel
The situation-based dispatch of organizations	Balancing the need for units with their availability
	Considering which other organizations to connect to the emergency call

The complexity of identifying chemical incidents

The ERC personnel assert that it is often difficult to gain situation awareness about whether a chemical incident is actually in progress, what kind of chemicals are involved, how dangerous the situation is, and its potential consequences.

Perceiving the chemical incident and its severity

Chemical incidents were considered as rare but potentially severe events, varying from singular chemically injured persons to major chemical incidents. The ERC personnel considered major chemical incidents as likely, and recognised that they have to be prepared for them. They considered themselves reasonably well prepared, and asserted that chemical incidents were not more difficult to manage than other incidents. However, participants stated that they became more focused and had to think outside the box when such incidents occurred, especially if they are unsure about the chemical reactions taking place, or in particularly difficult situations such as fires or incidents at sea.

Because of the rarity of chemical incidents, the ERC personnel stated that there was a risk that they would not perceive that a given incident indeed was chemical in nature. Thus, in order to

For peer review only - http://bmjopen.bmj.com/site/about/guidelines.xhtml

not dismiss a dangerous situation, participants mentioned that they spend more time on incidents they perceived as vague than on obvious ones. Participants mentioned that they have certain trigger words for chemical incidents, which were emission, leakage, traffic incident, strong strange smell, if people are feeling sick without a clear cause, smoke or gas leaks, dripping sounds, that something looks strange, fires in industries or cars, or whether a heavy goods vehicle is involved in the incident.

Chemical incidents were also considered as vague incidents because the chemicals can be invisible, which makes it difficult to assess the volume of the release. Participants also mentioned the difficulties of understanding the chemical incident's potential to escalate and become more dangerous in a short time. For example, incidents can escalate if a fire starts or spreads, if there is a combination of cargo, if the chemical substance comes into contact with water, or if the incident has occurred on a heavily trafficked road.

Another kind of difficult situation entailed having to identify symptoms (such as affected breathing, cough, stinging eyes, nausea, headache and dizziness) as being caused by a chemical incident, because these are such common symptoms. Other ERC personnel considered that chemical incidents could be obvious - for example incidents with a heavy goods vehicle transporting dangerous chemicals. The ERC personnel considered that it depended on the situation if they clearly comprehended that it was a chemical incident or not - some callers may be very verbal and factual regarding relevant information, while others may be less communicative when anxious:

> *They called and said that a person had inhaled chlorine gas at an ice-hockey stadium, which is not strange, it can happen. It never emerged in the call that it was still leaking. The callers never mentioned this, they were mostly shocked. Then the ERC operator had to ask "is it still leaking?". It took about 20-30*

For peer review only - http://bmjopen.bmj.com/site/about/guidelines.xhtml

seconds to ask that question, because it didn´t emerge spontaneously. (Rescue service dispatcher)

Identifying chemical substances based on the situation

ERC personnel asserted that it was important to quickly identify the chemical substance or substances in play, as well as how dangerous it or they were, but also felt that this was not always easy. One ERC operator mentioned that the caller is their eyes on site. In order to assess the situation and find out which chemical is involved, there are a lot of questions the ERC operators could ask - for example, what does the smoke/substance look like, how does it smell or if observers can see any orange warning signs at heavy goods vehicles. The ERC operators acknowledged that they could be ignorant about chemicals and their effects, and could be in need of more information from the rescue service, the specialist emergency medical service for chemical incidents or the Swedish Poisons Information Centre. The rescue service dispatchers mentioned that they were knowledgeable about chemicals and could also search for more information in the chemical substance database.

If we receive a chemical formula that we don't recognise, we search the chemical substance database in order to be able to notify the rescue service when they are on their way. We get to know that the chemical substance can react with oxygen and can be ignited at 27°C and it is 22 °C outside. This means lives are at risk for those who are at the site of the incident. (ERC operator)

The ERC personnel felt that the general public had minimal knowledge about chemicals and thus preferred if professional personnel (e.g. heavy goods vehicle drivers and industry workers) made the emergency call. The professionals are trained in what to say - they know which chemical substances they handle, and are knowledgeable about which risks a given substance entails. In certain cases, e.g. if a driver is injured, the ERC personnel could call the

For peer review only - http://bmjopen.bmj.com/site/about/guidelines.xhtml

transportation business to ask what the heavy goods vehicle is transporting. However, during major incidents, e.g. major industry incidents, the ERC operators stated that it could be really difficult to gain information from the professional personnel. They mentioned that their first operational picture could change really quickly while the call progressed and they received more information.

The importance of ensuring the safety of citizens and personnel of emergency organizations

For peer review only

The ERC personnel stated that they have to attain situation awareness about the chemical incident in order to ensure the safety of both the citizens at the incident site and the emergency organization personnel that will arrive at the incident site.

Giving advice to the caller about safety

The ERC operators considered it difficult to give advice to callers during chemical incidents, but acknowledged that they had enough experience and common sense to give sound advice. Most of the time, operator advice was generic, but operators could also give advice from a chemical substance database or based on recommendations from the rescue service. Examples of advice were to move further away from the incident and be mindful of the direction of the wind, to evacuate buildings and wait for the rescue service to rescue trapped persons.

While the ERC operators volunteered that they want to receive as much information as possible, they still saw their prime responsibility as ensuring the safety of the caller. Thus, they have to be careful when asking callers to investigate the scene - for example, in case of missed warning signs. If there are others injured at the scene of the incident, they are a secondary priority if there is a risk of even more casualties. Operators usually had to be very straightforward with the callers in telling them to think of their own safety, leave injured

For peer review only - http://bmjopen.bmj.com/site/about/guidelines.xhtml

persons behind and advise them against running into dangerous situations, as well as in advising callers to keep distance while waiting for experts with appropriate equipment. Sometimes the caller wants to help but does not see the seriousness of the situation, and this might lead to an even worse situation, despite their good intentions.

> *When it comes to animals which may burn to death inside a barn or when there are still people left in a burning building, those scenarios are difficult, because people want to run in, most of them anyway. We want them to keep as far away as possible in order for them not to hurt themselves. (ERC operator)*

Considering the safety of the emergency organizations' personnel

The ERC personnel considered it important that they correctly inform the emergency organizations of the chemical incident. Despite not always knowing the chemical substance involved, participants verify that the emergency organizations perceive that it is a dangerous situation. Thus, ERC personnel would rather give a bit too much information than too little, so that the emergency organizations can make their own assessments of the situation. If ERC personnel have knowledge of the chemical substance involved, they can give relevant safety advice, for example on safety distances. When they do not know the chemical substance involved, the dispatchers considered it to be difficult to indicate to the emergency organizations that a chemical incident actually is in progress. If they do not know the chemical substance(s) involved, or have very little information about the incident, ERC personnel report that they will work as if the worst possible situation prevailed, and then (if called for) de-escalate the situation, based on information from the emergency organizations at the incident site.

> *Interviewer: If you had received information that it was hydrochloric acid, what decisions would you then have made?*

For peer review only - http://bmjopen.bmj.com/site/about/guidelines.xhtml

Rescue service dispatcher: I probably would not really have made any other decisions. However, I could have planned the rescue operation and what resources were necessary. I probably would not have needed as many resources, they could have extinguished the fire earlier at a closer range.

The situation-based dispatch of organizations

During chemical incidents, the ERC personnel need situation awareness in order to interpret the situation, as well as to inform and dispatch the required organizations. Participants believed that more resources are needed during chemical incidents than during other incidents, but also emphasise that limits on resources must also be taken into consideration.

Balancing the need for units with their availability

The dispatchers decide which emergency organization units should be dispatched to an incident based on the first incoming information from the callers, pre-made plans or directives, and the competences of the dispatched units (e.g., the specialist emergency medical service for chemical incidents). This means they have to dare to make quick decisions about how many units they should dispatch to the incident.

The rescue service would rather dispatch 10 times too many units than miss dispatching units when we could have saved someone. We are drilled that if we happen to dispatch two rescue service units too many, we can just withdraw them if they are not needed. (Rescue service dispatcher)

The dispatchers' decisions about the number of dispatched units differ depending on the situation - for example, the risk for further injuries, quantity of chemical substances involved, and type of environment. Participants assert that chemical incidents demand more units than other incidents - something which often requires that extra emergency medical service units

For peer review only - http://bmjopen.bmj.com/site/about/guidelines.xhtml

be sent, because there is a risk of injury to rescue service personnel. If a major incident is in progress, dispatchers will send all the available units in the region. However, the emergency medical service dispatchers considered they had to prioritise where they send their units, because they also have a responsibility to care for all other patients with severe injuries and illnesses. For example, in a city they may be able to send all available units to a chemical incident, but not in the countryside because of long distances.

The dispatchers report working with the resources they have available - for example, moving resources in the region to strategic places in order to cover the entire region. They can also collaborate with other regions in order to increase the available resources. The dispatchers will also have a dialogue with the emergency organizations at the incident site if more resources are needed and they can give suggestions of additional available resources. It is the incident commanders who decide which resources are needed based on the situation at the incident site, in relation to the available resources.

Considering which other organizations to connect to the emergency call

The ERC operators report that they quickly have to consider, contact and inform several organizations and chemical experts, apart from the rescue service and emergency medical dispatchers. For example, they report that there are specialised rescue services which are contacted if an incident happens within their area of responsibility, such as the sea, mountain, industry or airport rescue service. The ERC operators also described that they contact, ask for more information and inform the police response centre operators that dispatch police units to the chemical incident.

> *When I understand that it's an incident with several [people] exposed to the same substance, I contact the rescue service and the police. I ask them if they know anything and give them the information we have, that there are several*

For peer review only - http://bmjopen.bmj.com/site/about/guidelines.xhtml

persons that have been exposed and we suspect that it's this substance but we have no idea, but there are people that have been in a specific place and they start to show symptoms and something isn't right. (ERC operator)

Other relevant representatives the ERC personnel mentioned that they can consider contacting, including officials on call, the salvage officer and representatives from the Swedish Transport Administration. The officials on call are contacted for resource allocation and preparation of the hospitals in the region. The salvage officer works together with the rescue service to protect the environment - for example, by taking care of spilled chemicals and by documenting the incident. Representatives from the Swedish Transport Administration are contacted if the ERC personnel consider it important to block a road or reroute traffic. If there is a major chemical incident, they also contact the Swedish Civil Contingencies Agency, who inform the citizens of the chemical incident through an Important Public Announcement. This could for example happen if there is a risk to the health and safety of the citizens due to toxic smoke, or if an area is blocked off due to the risk of explosion.

DISCUSSION

This study aimed at examining the situation awareness of the personnel working at ERCs - how they perceive, comprehend, project and act during chemical incidents. The three categories of the results will be discussed further below.

Starting with the category of the complexity of identifying chemical incidents, the result show that it could take time to ascertain whether a situation was dangerous or had the potential to escalate. Prior research has also confirmed that ERC personnel can receive inadequate information or have difficulties in grasping the situation (24). During chemical incidents this can be especially true if the incident type is not covered by the emergency dispatch index interview guide, or if the information about the incident is fragmented and several callers have

For peer review only - http://bmjopen.bmj.com/site/about/guidelines.xhtml

to provide information for a full understanding of the situation, as reported in Westman et al. 2021(25). In situations having a clear emergency dispatch index, a swift response can be expected, thus dispatch indexes covering chemical incidents could be developed. As this result show the ERC personnel's gut feeling is central to picking up on cues that there has been a chemical incident. This is consistent with Leonardsen et al. (26), who state that ERC operators refer to a mental picture where they use their experience and intuition in order to decide how to properly deal with a call. Thus, the results agree with what Ek and Svedlund state (13), that the ERC operators have to balance between strictly using the interview guide and using common sense and intuition.

Prior literature also indicates that it is especially important to be vigilant during motor vehicle collisions, heavy goods vehicle incidents or structural fires, because for example the hazmat placard could have been damaged in the heavy goods vehicle crash (3). The result in this study shows that hazard plaques and environmental clues could be of great help, but sometimes the owner of the heavy goods vehicles involved was needed to be contacted if it was uncertain which chemical substance(s) was involved. This was sometimes complicated by the fact that the driver was unconscious and unable to answer any questions (3). A suggestion to further improve the knowledge of the ERC personnel on chemical incidents and implement lessons learned could be to develop emergency organization use of chemical incident databases, similarly to what is suggested in the chemical industry literature (27).

Another result of this study shows that it is easier to receive the emergency call from staff working at an affected site as the information from them are more accurate and clear and thus more useful for the operators than from other callers. This is somewhat similar to what Holmström, Kaminsky, Lindberg et al. (28) described, that it is more difficult to understand information and assess the severity of the symptoms from other callers e.g. relatives or strangers at an incident site, who do not have any background information about the sick or

For peer review only - http://bmjopen.bmj.com/site/about/guidelines.xhtml

injured persons. Thus, improving the public knowledge and ability to act during chemical incidents by performing pre-incident information campaigns about chemical incidents (29) could possibly lead to ERC personnel gaining more useful information from the public.

The category "The importance of ensuring the safety of citizens and personnel of emergency organizations" emphasises the importance of ERC personnel's situation awareness for their ability to give correct information and sound advice, because the chemical incident site may still be dangerous. Prior literature also states that it is important for ERC personnel to be able to assess risks, reassure and give the caller appropriate instructions and safety advice, as well as to gather and share information with the emergency organizations in order to prepare them - both tasks, when executed, will decrease the risks to all involved (16, 24, 25, 30). This is important because it is relatively common that the personnel of the emergency organizations are injured during chemical incidents (31). Thus, the ERC personnel of this study stated that it was important to inform the emergency organizations about the situation at the incident site, including information about the chemical substance if they knew it.

Further, prior literature has also pointed out that if the chemical substance in play is known, so is the route of exposure and the likelihood for secondary contamination of the emergency organization personnel, as well as the most effective method of protection and decontamination (3). Further, stressing the importance of correct information from the ERC personnel, Keene et al. 2022(32) states that there is a risk that the emergency organizations are misguided, because they form their impressions of the incident based on pre-arrival information rather that post-arrival information. This is also discussed in another study, which state that incomplete information from the ERC personnel could lead the emergency medical service units to misinterpret the situation and lead to wrong actions, which in turn leads to delayed care of those affected (33). Thus, the importance of the ERC personnel to ensure the safety of both citizens and emergency personnel cannot be stressed too much.

For peer review only - http://bmjopen.bmj.com/site/about/guidelines.xhtml

Roud et al. 2021has found that so-called reflection seminars can be helpful in focusing on unsolved problems in structures, behaviours and working methods, as well as in confirming existing knowledge and procedures (34). Such seminars may also build trust and collaboration between emergency organizations, and complement learning obtained from full-scale exercises (34). Thus, in order to decrease the risk of injuries to emergency personnel and citizens, it could potentially be of value to create forums for all responding organizations including the ERC personnel, where learning from each other about safety during potential chemical incidents could be discussed. Lessons learned could then be further inculcated and put into practice during full-scale exercises.

Finally, the importance of the situation-based dispatch of organizations was in this study confirmed by ERC personnel reports that they usually worked with a given understanding of which organizations and units needed to be informed and dispatched to the chemical incident. However, although the ERC personnel in this study stated that chemical incidents demanded a lot of units, they also had to take into consideration how many units they actually had available and prioritise between patients. This is consistent with what other authors state, that ERC personnel have to consider that there are limited resources and that they had to prioritise the calls in relation to other waiting calls (12, 13). Thus, also prior literature states that ERC personnel have to be aware of what is happening in the area regarding available resources and other ongoing incidents in order to send sufficient resources (7).

Another issue relating to the issues of limited resources is that chemical incidents can be perceived as vague, as shown in this study's results. One prior study has also found that sending resources to vague but urgent alarms could be problematic if the situation responded to was found to be non-urgent upon arrival (30). Thus, ERC personnel have to balance the need of resources with the availability of resources. Reflection, seminars could provide a

For peer review only - http://bmjopen.bmj.com/site/about/guidelines.xhtml

forum where ERC personnel can discuss resource management during chemical incidents with representatives from the emergency organizations.

Strengths and limitations

One strength of the study is that the participants come from different ERCs - thus, we can gain a geographically diverse understanding of the situation awareness of ERC personnel. One limitation is that the Swedish system is very much a diverse system when it comes to the roles and responsibilities and even education of the Emergency Response operators and emergency organizations' dispatchers. This may lead to a fragmented understanding of personnel's perspectives - perhaps, a greater number of interviews would have been necessary to cover more aspects of the study. The interviews were also performed via Zoom and Teams due to Covid-19, which can influence the quality of the interviews. The participants themselves could choose if they wanted to have a videocall or if they did not want their picture showing, in order for them to feel as comfortable as possible. However, all in all, the participants gave a varied understanding of how it is to work during chemical incidents and the interviews were rich and in general about one hour in length, which resulted in a lot of material to analyse. No limit was set for the period of time that had passed since the incident occurred as it is the content of the stories that responds to the purpose of the study.

Practical implications and recommendations for future research

This study explore the situation awareness of the ERC personnel and identified challenges are discussed. In order to further increase the situation awareness of ERC personnel, a practical implication of this study could be to further develop emergency dispatch index interview guides that covers this type of incidents, so that it is more likely that the ERC personnel ask the most efficient questions. This could be an area where more research and development are needed.

For peer review only - http://bmjopen.bmj.com/site/about/guidelines.xhtml

Further practical implications could be to add a chemical incident database to the chemical substance database. In this database, all incidents could be documented together with their responses and lessons learned and continually discussed in reflection seminars for ERC and emergency organization personnel. Further research is needed of how such critical reflections could be implemented in practice and further improved during e.g. joint training and full-scale exercises. Such a database could theoretically also be a foundation to calculate resource allocation to such an incident.

CONCLUSION

This study explore how ERC personnel attain accurate situation awareness of what is happening during potentially dangerous chemical incidents in order to be able to notify, inform and dispatch the correct resources. During chemical incidents there are several dichotomies. One is between the ERC personnel wanting as much information as possible for everyone's safety, also for emergency organizations, and their responsibility for the safety of the caller. This is not always a straightforward task, especially since chemical incidents can be considered vague. Another dichotomy is between utilizing emergency dispatch index interview guides versus the ERC personnel trusting their gut feeling. Even if it would be possible to cover all probable chemical incidents in the emergency dispatch index interview guide it would still be necessary for the ERC personnel to trust their gut in the first place to even be able to identify the chemical incident. Thus, more research is needed to further explore these dichotomies.

Acknowledgement The authors would like to thank all participants for their contribution to the research. The authors would also like to thank professor Britt-Inger Saveman for helpful suggestions during the conception of the study and interview guide and recruitment of

For peer review only - http://bmjopen.bmj.com/site/about/guidelines.xhtml

participants. The authors would also like to thank Raimo Lindgren for help with performing four of the interviews. We would also like to thank Johanna Björnstig for writing the transcriptions of the interviews.

Contributors SK and LG conceived and designed the study. SK interviewed all but four of the participants, which was performed by a research assistant. SK performed the first analysis and wrote the first draft of the manuscript both which was concurrently discussed with LG and revised. The final manuscript is approved by both SK and LG. LG is the guarantor.

Patient and public involvement in research Patients or the public were not involved in the design, conduct, reporting or dissemination of this research.

Ethics approval The study's objectives and methods are not covered by the Swedish Act concerning the Ethical Review of Research Involving Humans. Thus, approval from the Swedish Ethical Review Authority for this study has not been sought.

Competing interests None declared

Funding The research was funded by the Swedish National Board of Health and Welfare, grant number N/A.

Data availability statement All data relevant to the study are included in the article or uploaded as supplementary information.

Patient consent for publication Not applicable

REFERENCES

1. World Health Organization. Manual for the public health management of chemical incidents. Geneva:2009.

For peer review only - http://bmjopen.bmj.com/site/about/guidelines.xhtml

2. Newmark J. Clinical aspects of large-scale chemical events. In: Koenig KL, Schultz CH, eds. Koenig and Schultz's disaster medicine comprehensive principles and practice. 2nd edition. Cambridge, Cambridge University Press: 2016:499–521.

3. Lim HC, Tan HH. Hazardous material, toxic, and industrial events. In: Koenig KL, Schultz CH, eds. Koenig and Schultz's disaster medicine comprehensive principles and practice. 2nd edition. Cambridge, Cambridge University Press: 2016:579–599.

4. United Nations Office for the Coordination of Humanitarian Affairs (OCHA). Lebanon: Beirut Port Explosions Situation Report No.1 (As of 5 August 2020) [online]. 2020. https://reliefweb.int/report/lebanon/lebanon-beirut-port-explosions-situation-report-no-1-5-august-2020-enar (accessed 29 November 22).

5. TT. Ammoniakutsläpp i Helsingborg (Swe). Ammonia emissions in Helsingborg. [online] 2018. from https://www.svt.se/nyheter/inrikes/ammoniakutslapp-i-helsingborg. (accessed 29 November 2022).

6. Lindberg J. Brand på Northvolt: Flera personer till sjukhus (Swe) [online]. Fire at Northvolt: Several people taken to hospital. Västerbottenskuriren. 2022. https://www.vk.se/2022-10-07/brand-pa-northvolt-flera-personer-till-sjukhus (accessed 28 November 2022).

7. Norri-Sederholm T, Seppälä J, Saranto K, et al. Information flow and situational awareness in emergency medical dispatch. International Journal of Networking and Virtual Organisations. 2016;16(1):72–85. doi: https://doi.org/10.1504/IJNVO.2016.075134

8. Swedish Government Official Reports. SOU 2018:28 En nationell alarmeringstjänst - för snabba säkra och effektiva hjälpinsatser (Swe). SOU 2018:28 A national emergency response center - for rapid, secure and effective rescue operations. Ministry of Justice, Stockholm:2018.

For peer review only - http://bmjopen.bmj.com/site/about/guidelines.xhtml

For peer review only

9. Magnussen LI, Carlstrøm E, Berge AK, et al. Help we are sinking! Stories from Norwegian dispatch centers on decision-making in unfamiliar and ambiguous situations. Journal of Emergency Management. 2018;16(4):245–254. doi: https://doi.org/10.5055/jem.2018.0373

10. Blandford A, Wong BLW. Situation awareness in emergency medical dispatch. International Journal of Human-Computer Studies 2004;61(4):421–452. doi: https://doi.org/10.1016/j.ijhcs.2003.12.012

11. Endsley MR. Toward a theory of situation awareness in dynamic systems. Human Factors: The Journal of the Human Factors and Ergonomics Society 1995;37(1):32–64. doi: https://doi.org/10.1518/001872095779049543

12. Leonardsen A-C, Ramsdal H, Olasveengen TM, et al. Exploring individual and work organizational peculiarities of working in emergency medical communication centers in Norway – a qualitative study. BMC Health Services Research. 2019;19:545 https://doi.org/10.1186/s12913-019-4370-0

13. Ek B, Svedlund M. Registered nurses' experiences of their decision-making at an Emergency Medical Dispatch Centre. Journal of Clinical Nursing. 2015;24(7-8):1122–1131, doi: https://doi.org/10.1111/jocn.12701

14. Torlén Wennlund K, Kurland L, Olanders K, et al. Emergency medical dispatchers' experiences of managing emergency calls: a qualitative interview study. BMJ Open. 2022;12:e059803. doi: 10.1136/bmjopen-2021-059803

15. Rispens JR, Jones SA, Clemmons NS, et al. Anhydrous ammonia chemical release - Lake County, Illinois, April 2019. Morbidity and Mortality Weekly Report. 2020;69(4):109–113. doi: 10.15585/mmwr.mm6904a4

For peer review only

For peer review only - http://bmjopen.bmj.com/site/about/guidelines.xhtml

16. Gardett I, Scott G, Broadbent M, et al. Situational awareness in emergency medical dispatch: An obervation study and proposed model. Annals of Emergency Dispatch and Response 2019;7(2):16–22.

17. The National Board of Health and Welfare in Sweden. Händelser med kemikalier - Kunskapsunderlag för hälso- och sjukvården (Swe). Chemical incidents - Document of knowledge for the health care. Stockholm: 2009.

18. Uppsala University Hospital. Sjukvårdens larmcentral (Swe). Emergency medical dispatch center [online]. 2020. https://www.akademiska.se/for-vardgivare/verksamhetsomraden/ambulanssjukvard/larmcentral/ (accessed 28 November 2022).

19. Graneheim UH, Lundman B. Qualitative content analysis in nursing research: concepts, procedures and measures to achieve trustworthiness. Nurse Education Today 2004;24(2):105–112. doi: https://doi.org/10.1016/j.nedt.2003.10.001

20. Graneheim UH, Lindgren B-M, Lundman B. Methodological challenges in qualitative content analysis: A discussion paper. Nurse Education Today 2017;56:29–34. doi: https://doi.org/10.1016/j.nedt.2017.06.002

21. Lindgren B-M, Lundman B, Graneheim UH. Abstraction and interpretation during the qualitative content analysis process. International Journal of Nursing Studies. 2020;108:103632. doi: https://doi.org/10.1016/j.ijnurstu.2020.103632

22. World Medical Association. World Medical Association Declaration of Helsinki - Ethical principles for medical research involving human subjects [online]. 2013. from https://www.wma.net/policies-post/wma-declaration-of-helsinki-ethical-principles-for-medical-research-involving-human-subjects/ (accessed 29 November 2022).

For peer review only

For peer review only - http://bmjopen.bmj.com/site/about/guidelines.xhtml

23. Swedish Code of Statutes. Lag (SFS 2003:460) om etikprövning av forskning som avser människor (Swe). Law concerning the ethical review of research involving humans. The ministry of education and research. Stockholm:2003.

24. Forslund K, Kihlgren A, Kihlgren M. Operators' experiences of emeregency calls. Journal of Telemedicine and Telecare 2004;10(5):290–297. doi: https://doi.org/10.1258/1357633042026323

25. Westman A, Saveman B-I, Björnstig U, et al. Mobilisation of emergency services for chemical incidents in Sweden - a multi-agency focus group study. Scandinavian Journal of Trauma, Resuscitation and Emergency Medicine. 2021;29:99. doi: https://doi.org/10.1186/s13049-021-00910-5

26. Leonardsen AC, Ramsdal H, Olasveengen TM, et al. Exploring individual and work organizational peculiarities of working in emergency medical communication centers in Norway – a qualitative study. BMC Health Services Research. 2019;19:545 doi: https://doi.org/10.1186/s12913-019-4370-0

27. Wood MH, Koutelos K, Hailwood M, et al. Learning lessons from chemical incidents – What's stopping us and how we can make it happen. Chemical Engineering Transactions 2022;90:685–690. doi: https://doi.org/10.3303/CET2290115

28. Holmström IK, Kaminsky E, Lindberg Y, et al. The perspectives of Swedish registred nurses about managing difficult calls to emergency medical dispatch centres: a qualitative descriptive study. BMC Nursing 2021;20:150. doi: https://doi.org/10.1186/s12912-021-00657-5

29. Carter H, Weston D, Symons C et al. Public perceptions of pre-incident information campaign materials for the initial response to a chemical incident. Disaster Prevention and Management 2019;28(5):565–584. doi: https://doi.org/10.1108/DPM-10-2018-0342

For peer review only - http://bmjopen.bmj.com/site/about/guidelines.xhtml

30. Palsgaard Møller T, Gamst Jensen H, Viereck S, et al. Medical dispatchers' perception of the interaction with the caller during emergency calls – a qualitative study. Scandinavian Journal of Trauma, Resuscitation and Emergency Medicine. 2021;29:45 doi: https://doi.org/10.1186/s13049-021-00860-y

31. Melnikova N, Wu J, Yang A, et al. Acute chemical incidents with injured first responders, 2002-2012. Disaster Medicine and Public Health Preparedness. 2018;12(2):211–221. doi: https://doi.org/10.1017/dmp.2017.50

32. Keene T, Pammer K, Lord B, et al. Dispatch information affects diagnosis in paramedics: an experimental study of applied dual-process theory. International Journal of Emergency Services 2022;11(2):277–291. doi: https://doi.org/10.1108/IJES-06-2021-0039

33. Hernborg MN, Carlström ED, Berlin JM. First initiatives in prehospital care – Basing assessments on incomplete preliminary information. Journal of Nursing Education and Practice 2020;10(6):47–54. doi: https://doi.org/10.5430/jnep.v10n6p47

34. Roud E, Gausdal AH, Asgary A, et al. Outcome of collaborative emergency exercises: Differences between full-scale and table-top exercises. Journal of Contingencies and Crisis Management 2021;29(2):170–184. doi: https://doi.org/10.1111/1468-5973.12339

For peer review only

For peer review only - http://bmjopen.bmj.com/site/about/guidelines.xhtml

Interview guide: Situation awareness, Emergency Response Centre personnel

General information about the participant:

- How old are you?
- How many years have you worked at the Emergency Response Centre?
- Are you an operator/rescue service dispatcher/emergency medical service dispatcher?
- Have you previously worked with something that can be relevant considering that the interview is about chemical incidents? If yes – for how many years?
- What kind of education do you have?

Interview questions:

1. Can you describe a situation where you received an emergency call regarding an incident, immediately or later classified as a chemical incident, where there was a risk of people being injured or dying?
    a. What made you understand that it was more than an ordinary incident – an incident with chemicals involved when there was a risk of casualties?
        i. Was it something the caller said?
        ii. How did you find out the extent of the incident, for example if there were several people injured?
        iii. Was it evident from the start that it was a chemical incident with a risk for casualties?
    b. When you understood that it was a chemical incident with a risk for casualties, did you have access to an interview guide or other guide?
        i. What made you choose a specific interview guide?
            1. Do you ever need ask other questions than those in the interview guide? If yes – why?
            2. If you needed to ask other questions than those in the interview guide, whom did you ask and what did you ask?
        ii. Did you receive information about which chemical substance was involved?
            1. How do you generally receive information about which chemical substance is involved? / How do you usually do to receive information about which chemical substance is involved?
            2. Depending on the chemical substance, how did you have to adjust your questions in order to know how you should act?
    c. Did you consider that the incident could become dangerous for more people? /escalate?
        i. Why? / why not?
        ii. How did you think the situation could develop?
            1. Before the emergency organizations arrived?
                a. Do you manage the emergency calls about chemical incidents differently depending on the lead time, e.g., if

the incident is in a sparsely populated area or quite close by?

2. When the emergency organizations were at the incident site?
3. When the emergency organizations had left the incident site? (Is there a continued risk?)
    a. During major chemical incidents, the incident site can be polluted/unsafe for a long time - do you have continual preparedness for several people being injured at the same incident site?

d. What decisions did you make?
    i. What information did you give to the caller, what should he/she do?
        1. What do you think of the dilemma of safety versus saving lives?
    ii. How did you know which resources needed to be dispatched?
        1. Are there dispatch plans?
    iii. Is there a particular order in which you contact the emergency organizations? If yes – does this differ depending on if it is a chemical incident with the risk for casualties?
    iv. How do you inform the emergency organizations that it is a chemical incident with the risk for casualties?
    v. Which information do you expect the emergency organizations at the incident site to send to you?
    vi. How does the information you receive from the emergency organizations at the incident site influence your actions?
    vii. Do the decisions you take differ depending on the chemical substance involved?
    viii. Do your decisions differ depending on if you suspect an antagonistic incident and not a regular incident?

General questions to ask after the participants have described their experiences

1. Is there anything regarding emergency calls about chemical incidents with the risk for casualties that you consider challenging? - What? Why? / Why not?
    a. Do you need the support of a back office/ other support during chemical incidents? Do you sit together?
    b. Are there occasions when you feel that the incident is worse than what the caller thinks? – In that case, what do you do?
2. Are there situations when you more often consider that it can be a chemical incident with the risk for casualties?
    a. If an alarm comes from a Seveso facility, for example, does this influence how you think about the incident?
        i. Is there any extra information that you would ask for?
    b. Depending on if it is a citizen that makes the emergency call, a professional working at a Seveso facility, or a heavy goods vehicle driver, do you think differently?
    c. If an electric bus start burning, how do you proceed?
        i. Are there special tasks you need to perform?
        ii. How do you think the situation could develop if this happened in a tunnel/residential area?

For peer review only

3. If you receive an emergency call that suggests that the person's symptoms could be a consequence of a chemical incident, which information would you need to be certain?
    a. If several emergency response centre operators receive calls about persons with the same symptoms, how would you understand if it was the same chemical incident?
    b. During chemical incidents the symptoms does not always present at once. Do you usually take this into consideration when you make decisions?
4. How do you work with the operator/dispatchers during joint emergency calls during chemical incidents with the risk for casualties? Is there any difference compared to other major incidents?
    a. How do you ensure that all persons in the joint emergency call have the same information about the chemical incident?
5. What responsibility do you have when you have dispatched the emergency organization units and they have arrived at the incident site?
    a. Do you need to be prepared to adjust the emergency organization units depending on how serious the incident is and how many injured there are?
    b. If, during a chemical incident, people in the emergency organization units would become injured due to lacking information, what would you think of such an incident?
6. Is there anything which we have not talked about that you want to bring up? Do you have any questions?

For peer review only

COREQ (COnsolidated criteria for REporting Qualitative research) Checklist

A checklist of items that should be included in reports of qualitative research. You must report the page number in your manuscript where you consider each of the items listed in this checklist. If you have not included this information, either revise your manuscript accordingly before submitting or note N/A.

Topic	Item No.	Guide Questions/Description	Reported on Page No.
**Domain 1: Research team and reflexivity**			
Personal characteristics			
Interviewer/facilitator	1	Which author/s conducted the interview or focus group?	
Credentials	2	What were the researcher's credentials? E.g. PhD, MD	
Occupation	3	What was their occupation at the time of the study?	
Gender	4	Was the researcher male or female?	
Experience and training	5	What experience or training did the researcher have?	
Relationship with participants			
Relationship established	6	Was a relationship established prior to study commencement?	
Participant knowledge of the interviewer	7	What did the participants know about the researcher? e.g. personal goals, reasons for doing the research	
Interviewer characteristics	8	What characteristics were reported about the inter viewer/facilitator? e.g. Bias, assumptions, reasons and interests in the research topic	
**Domain 2: Study design**			
Theoretical framework			
Methodological orientation and Theory	9	What methodological orientation was stated to underpin the study? e.g. grounded theory, discourse analysis, ethnography, phenomenology, content analysis	
Participant selection			
Sampling	10	How were participants selected? e.g. purposive, convenience, consecutive, snowball	
Method of approach	11	How were participants approached? e.g. face-to-face, telephone, mail, email	
Sample size	12	How many participants were in the study?	
Non-participation	13	How many people refused to participate or dropped out? Reasons?	
Setting			
Setting of data collection	14	Where was the data collected? e.g. home, clinic, workplace	
Presence of non-participants	15	Was anyone else present besides the participants and researchers?	
Description of sample	16	What are the important characteristics of the sample? e.g. demographic data, date	
Data collection			
Interview guide	17	Were questions, prompts, guides provided by the authors? Was it pilot tested?	
Repeat interviews	18	Were repeat inter views carried out? If yes, how many?	
Audio/visual recording	19	Did the research use audio or visual recording to collect the data?	
Field notes	20	Were field notes made during and/or after the inter view or focus group?	
Duration	21	What was the duration of the inter views or focus group?	
Data saturation	22	Was data saturation discussed?	
Transcripts returned	23	Were transcripts returned to participants for comment and/or	

For peer review only

Topic	Item No.	Guide Questions/Description	Reported on Page No.
		correction?	
**Domain 3: analysis and findings**			
Data analysis			
Number of data coders	24	How many data coders coded the data?	
Description of the coding tree	25	Did authors provide a description of the coding tree?	
Derivation of themes	26	Were themes identified in advance or derived from the data?	
Software	27	What software, if applicable, was used to manage the data?	
Participant checking	28	Did participants provide feedback on the findings?	
Reporting			
Quotations presented	29	Were participant quotations presented to illustrate the themes/findings? Was each quotation identified? e.g. participant number	
Data and findings consistent	30	Was there consistency between the data presented and the findings?	
Clarity of major themes	31	Were major themes clearly presented in the findings?	
Clarity of minor themes	32	Is there a description of diverse cases or discussion of minor themes?	

Developed from: Tong A, Sainsbury P, Craig J. Consolidated criteria for reporting qualitative research (COREQ): a 32-item checklist for interviews and focus groups. *International Journal for Quality in Health Care*. 2007. Volume 19, Number 6: pp. 349 – 357

Once you have completed this checklist, please save a copy and upload it as part of your submission. DO NOT include this checklist as part of the main manuscript document. It must be uploaded as a separate file.

For peer review only
